# Impaired generation of new subcategories and switching in a semantic verbal fluency test in older adults with mild cognitive impairment

**DOI:** 10.3389/fnagi.2014.00141

**Published:** 2014-07-01

**Authors:** Laiss Bertola, Maria Luiza Cunha Lima, Marco A. Romano-Silva, Edgar N. de Moraes, Breno Satler Diniz, Leandro F. Malloy-Diniz

**Affiliations:** ^1^Laboratory of Clinical Neuroscience Investigations, Faculty of Medicine, Federal University of Minas GeraisBelo Horizonte, Brazil; ^2^National Institute of Science and Technology in Molecular Medicine, Faculty of Medicine, Federal University of Minas GeraisBelo Horizonte, Brazil; ^3^School of Linguistics, Federal University of Minas GeraisBelo Horizonte, Brazil; ^4^Mental Health Department, Faculty of Medicine, Federal University of Minas GeraisBelo Horizonte, Brazil; ^5^Medical Clinic Department, Faculty of Medicine, Federal University of Minas GeraisBelo Horizonte, Brazil

**Keywords:** semantic verbal fluency, clustering, switching, subcategories, mild cognitive impairment, Alzheimer's disease

## Abstract

The semantic verbal fluency task is broadly used in the neuropsychological assessment of elderly subjects. Even some studies have identified differences in verbal fluency clustering and switching measures between subjects with normal aging and a clinical condition such as mild cognitive impairment (MCI) and Alzheimer's disease, the results are not always consistent. This study aimed to compare clustering and switching measures of an animal's semantic verbal fluency task among normal controls (NC, *n* = 25), amnestic mild cognitive impairment (aMCI; *n* = 25), amnestic multiple domain Mild Cognitive Impairment (a+mdMCI; *n* = 25) and Alzheimer's disease (AD; *n* = 25) Brazilian subjects. The analyses were executed considering three (unifying the MCI subtypes) and four groups. As the data were not normally distributed, we carried out non-parametric tests (Kruskal-Wallis and Mann-Whitney tests) to evaluate the differences in performance in the measures of the verbal fluency test among the groups. The comparison demonstrated that the groups differed in the total of correct words produced, number of clusters and switching but the measure of new subcategories was the only with significant difference among the NC and all the clinical groups. The measure of new subcategories is the number of original subcategories inside the higher category of animals that the subject produced, such as farm, domestic, African animals. Our results indicate that semantic memory impairment is a visible and recent deficit that occurs even in non-demented subjects with very MCI and the implications of these findings are discussed.

## Introduction

Mild cognitive impairment (MCI) is common among older adults with prevalence estimates ranging from 3 to 42% (Yesavage et al., 2002; Ward et al., 2012), and subjects with MCI have increased risk of developing dementia (Han et al., 2012). The gold standard for the identification of MCI usually relies on comprehensive neuropsychological assessment. Nonetheless, neuropsychological assessments are expensive, time-consuming, need highly trained professionals to do and, thus, may not be readily available in clinical practice. Cognitive screening tests are widely available, have low costs and need no specialized training for its correct administration and interpretation of results. Though being routinely used in clinical practice to evaluate subjects with cognitive complaints, they are not sensitive to identify mild cognitive deficits (Diniz et al., 2007). As a consequence many older adults may not be correctly identified as MCI delaying the diagnosis until they reach the threshold for dementia.

Semantic verbal fluency (SVF) tests are one of the most common cognitive screening used in clinical and research settings. This task requires the initialization of a specific verbal behavior, the search for specific verbal information, and self-monitoring to avoid mistakes and repetitions. Additionally, it requires the availability of a specific semantic knowledge (Unsworth et al., 2011). The traditional scoring system is the sum of correct words produced within one minute (Strauss et al., 2006). The scores on this task has a good sensitivity and specificity to discriminate older adults with no cognitive impairment from those with dementia (Radanovic et al., 2009). Despite not useful to differentiate MCI from AD patients (Radanovic et al., 2009), lower scores on SVF is a predictor of progression from MCI to AD (O'Dowd et al., 2004; Saxton et al., 2004; Amieva et al., 2005; Cottingham and Hawkins, 2009).

In addition to the traditional score, other information provided by the SVF can add valuable information about cognitive status of an individual. Clustering is the grouping of words belonging to the same subcategory and is a measure of semantic memory knowledge. Switching is the change of subcategory across all possibilities and measure the efficiency to retrieve the stored information (i.e., measure of executive functioning). Previous studies found that AD patients produced smaller clusters and switched less frequently from subcategories than normal controls (Troyer et al., 1998; Murphy et al., 2006; Fagundo et al., 2008). Recently, Price et al. (2012) showed that subjects with that amnestic MCI produced, smaller cluster sizes and fewer new subcategories when compared to controls, but the frequency of switches was not significantly different. Murphy et al. (2006) reported similar results, except that amnestic MCI subjects did not differ in the mean cluster size from the healthy subjects.

As SVF is commonly used in clinical practice and the analysis of other aspects of this task can add important information on the cognitive status of a patient, we aim to evaluate whether measures of clustering, switching, and number of subcategories can help to distinguish MCI and AD patients from healthy older adults with no cognitive decline. In addition, we aim to compare these measures between subgroups of MCI subjects. We hypothesize that patients with MCI and AD will show significant differences in these measures in comparison to healthy controls; and that there is gradual decline in these measures as the subjects progress from amnestic single domain MCI to amnestic multiple domain MCI, and finally to AD.

## Materials and methods

### Participants and assessment

One hundred older adults were included in this study. These participants were referred to a secondary outpatient geriatric unit for a comprehensive geriatric clinical assessment. All participants underwent a comprehensive clinical interview done by a geriatrician, and evaluated with a standardized neuropsychological protocol (de Paula et al., 2013). In brief, this protocol included the tests for the principal cognitive domains. General cognitive status (Mini Mental State Exam), executive functions (Frontal Assessment Battery, Letter Fluency of S and Digit Span), visioconstructional ability (Stick Design Test and Clock Drawing Test), episodic memory (Rey Auditory Verbal Learning Test), semantic memory [Naming Test (TN-LINC) and Category Verbal Fluency (Fruits)], and language (Token Test). The caregivers answered the General Activities of Daily Living Scale (GADL) that evaluates performance on activities of daily living (de Paula et al., 2014).

The neurocognitive status of each participant was adjudicated taking into account all information from the clinical and neuropsychological assessments. The participants were classified into three groups: normal controls (*n* = 25), MCI (*n* = 50), and mild AD (*n* = 25). MCI was diagnosed according to the Mayo Clinic Criteria (Petersen et al., 2001); AD was diagnosed according to the NINCDS-ADRDA criteria (McKhann et al., 1984). The normal control (NC) group included older adults without cognitive complains and scores above −1.5 SD of the mean according to local norms, adjusted for age and educational level.

The MCI participants was further subdivided into two groups: amnestic single domain—aMCI (*n* = 25) and amnestic multiple domain—a+mdMCI (*n* = 25) (Petersen, 2004). Amnestic MCI is defined by significant memory impairment and normal performance on other cognitive domains, no evidence of impairment in activities of daily living and preserved global cognition (Petersen et al., 2001). Amnestic multiple domain MCI is defined by significant memory impairment and of one or more additional cognitive domains (e.g., executive function, language), no evidence of impairment in activities of daily living and preserved global cognition. The local Ethics Committee approved this study and all participants and their families gave written consent.

### Verbal fluency test

All participants did the category verbal fluency test (animals). They were asked to say names of animals within 1 minute, and were advised to not repeat already spoken animals. All the words were recorded, including repetitions and errors (when other words that not animals were spoken).

#### Standard scores

The scoring procedure included the number of correct words, excluding number of errors (occurrence of words that do not refer to any animal) and number of repetitions (animals that were spoken more than once).

#### Clustering and switching scores

The scores for clustering and switching were obtained according to Troyer et al. (1997). Clusters were formulated according to the shared attributes between animals (e.g., farm animals, pet animals, zoo), or when an animal appeared alone. For example, the following animals compose one cluster: cow, pig, horse (farm animals). The cluster sizes were computed after a second word of the same subcategory if generated in sequence (cluster size = total of animals in a given cluster −1). For example, cluster with two words receive a size score of 1, with 3 words a size score of 2. If the word appeared alone, this cluster receives a size score of 0. The mean cluster size is the sum of all clusters sizes generated (including single words, repetitions, and errors) divided by the number of clusters. Switching is the number of changes in cluster generation during the task (Troyer et al., 1997). For example, a subject can say: cow, pig, horse (farm animals), whale, and fish (see animals). This subject produced one switching since he changed between the clusters of farm animals to a cluster of sea animals. We also included the measures of new subcategories (number of original subcategories produced, excluding reoccurrence of animals belonging to the same subcategory, but produced in a non-sequence way), as suggested by March and Pattison (2006). In the given example the subject produced two original subcategories. Other subject could saw: horse, cow, dog, cat, and pig. These second example shows that the subject first produced a cluster of farm animals (horse and cow), second a cluster of domestic animals (dog, cat), and third another cluster of farm animals (pig). In total these subject produces only two new subcategories of animals once he produced twice clusters of farm animals.

Additionally we proposed the scoring of the number of effective clusters that were developed, in which the subject produced more than one exemplar for that subcategory (horse and cow, instead of only saying horse), and the mean size of these developed clusters.

### Statistical analysis

We did Kolmogorov-Smirnov tests to evaluate the pattern distribution of the data. As the data were not normally distributed (data not shown), we carried out non-parametric tests (Kruskal-Wallis and Mann-Whitney tests) to compare for differences in the performance in the neuropsychological tests and measures of the verbal fluency test between groups. We also calculated the corresponding effect size (r) for each comparison. Chi-square tests were performed to analyze differences in the distribution of dichotomous variables between the groups. Statistical significance was set according to the Holm-Bonferroni correction for multiple comparisons. All analyses were performed with the Statistical Package for Social Science (SPSS), v.21 for Windows (IBM Corp. Released, 2012).

We carried out the same set of analysis dividing the MCI group according MCI subtype (groups: normal control, aMCI, a+mdMCI, AD).

## Results

### NC, MCI, AD

The AD, MCI, and control participants did not differ in most demographic data, except that the normal control group showed a significant higher frequency of women according to the Chi-square test (*X*^2^ = 6.76, *df* = 2, *p* = 0.009;). Participants with AD had worse performance in all neuropsychological measures and activities of daily living (Table 1). Participants with MCI had intermediate performance between AD and controls (Table 1).

**Table 1:** **Demographic description of NC, MCI, and AD groups**.

	**NC**		**MCI**		**AD**		**K–W**	
	**Median**	**Q1–Q3**	**Median**	**Q1–Q3**	**Median**	**Q1–Q3**	**H(2)**	***p***
Age	76	(70–81.5)	76	(70.5–81)	78	(67–81.5)	0.04	0.979
Education	4	(3–4)	4	(2–4)	4	(2.5–4)	0.34	0.840
GADL	26	(26–26)	26	(25–26)	20	(17.5–22)	62.74	**0.000^†^**
MMSE	27	(23.5–29)	25	(23–27)	20	(17–23.5)	29.18	**0.000^††^**

GADL, General Activities of Daily Living Scale; MMSE, Mini Mental State Exam.

† NC < MCI < AD;

†† *NC > MCI > AD*.

Table 2 shows the data from SVF variables. The groups significantly differed in total of words, number of clusters, new subcategories, and switching (Table 2).

**Table 2:** **Verbal fluency measures description of NC, MCI, and AD groups**.

	**NC**		**MCI**		**AD**		**K–W**		**Pairwise comparison^†^**
	**Median**	**Q1–Q3**	**Median**	**Q1–Q3**	**Median**	**Q1–Q3**	**H(2)**	***P***	
Corrects	14	(12–15.5)	11	(9.75–14)	9	(7–10.5)	26.28	**0.000**	NC > MCI > AD
Number of clusters	8	(6.5–9)	6	(4–8.25)	5	(4–6.5)	11.70	**0.003**	NC > AD
Mean clusters size	0.78	(0.50–1.14)	0.82	(0.52–1.47)	0.75	(0.5–1)	0.64	0.724	
Developed clusters	3	(2–4.5)	3	(2–3)	3	(2–3)	4.45	0.108	
Mean developed clusters size	1.80	(1.55–2.41)	1.71	(1.31–2.81)	1.33	(1–2.16)	3.81	0.148	
New subcategories	6	(5–7)	4	(3–6)	3	(2–4.5)	20.01	**0.000**	NC > MCI = AD
Switching	7	(5.5–8)	5	(3–8)	4	(3–5.5)	11.18	**0.004**	NC > AD

† *Mann-Whitney of verbal fluency measures between NC, MCI, and AD groups (significant p-value < 0.016 after Holm-Bonferroni correction for multiple analysis). Bold values of p means that the p-value was statistically significant*.

Pairwise comparison using the Mann-Whitney non-parametric test showed that all the three groups differed from each other on the number of correct words produced (Table 2). The NC group produced more new subcategories than MCI and AD, but there were no difference in these measure between the clinical groups (*p* = 0.072;). The NC group performed significantly better than AD also for number of clusters and switching (Table 2).

### NC, aMCI, a+mdMCI, AD

After we divided the MCI group in aMCI and a+MCI we found a significant difference between the groups in in daily living activities and general cognitive status (Table 3), and in the SVF measures of correct words, number of clusters, new subcategories, and switching (Table 4).

**Table 3:** **Demographic description of NC, aMCI, a-mdMCI, and AD groups**.

	**NC**		**aMCI**		**a+mdMCI**		**AD**		**K–W**	
	**Median**	**Q1–Q3**	**Median**	**Q1–Q3**	**Median**	**Q1–Q3**	**Median**	**Q1–Q3**	**H(3)**	***p***
Age	76	(70–81.5)	75	(70–79.5)	79	(71–81)	78	(67–81.5)	1.18	0.756
Education	4	(3–4)	4	(1.5–5)	3	(2–4)	4	(2.5–4)	2.55	0.466
GADL	26	(26–26)	26	(25–26)	25	(24–26)	20	(17.5–22)	63.67	**0.000^†^**
MMSE	27	(23.5–29)	26	(23–28)	24	(22.5–26)	20	(17–23.5)	31.57	**0.000^††^**

GADL, General Activities of Daily Living Scale; MMSE, Mini Mental State Examination.

† NC < aMCI; a+mdMCI < AD;

†† *NC > a+mdMCI > AD; aMCI > AD*.

**Table 4:** **Verbal fluency measures description of NC, aMCI, a-mdMCI, and AD groups**.

	**NC**		**aMCI**		**a+mdMCI**		**AD**		**K-W**		**Pairwise comparison^†^**
	**Median**	**Q1–Q3**	**Median**	**Q1–Q3**	**Median**	**Q1–Q3**	**Median**	**Q1–Q3**	**H(3)**	***P***	
Corrects	14	(12–15.5)	11	(10–14.5)	11	(9–13.5)	9	(7–10.5)	27.24	**0.000**	NC > a+mdMCI, AD; aMCI > AD
Number of clusters	8	(6.5–9)	8	(4.5–9)	6	(4–7)	5	(4–6.5)	14.30	**0.003**	NC > a+mdMCI, AD
Mean clusters size	0.78	(0.50–1.14)	0.67	(0.41–1.32)	1	(0.65–1.63)	0.75	(0.5–1)	2.36	0.501	
Developed clusters	3	(2–4.5)	3	(2–3.5)	3	(2–3)	3	(2–3)	4.62	0.202	
Mean developed clusters size	1.80	(1.55–2.41)	2	(1–3.41)	1.67	(1.36–2.41)	1.33	(1–2.16)	3.81	0.282	
New subcategories	6	(5–7)	4	(3–6)	4	(3–5)	3	(2–4.5)	20.82	**0.000**	NC > aMCI; a+mdMCI; AD
Switching	7	(5.5–8)	7	(3.5–8)	5	(3–6)	4	(3–5.5)	13.07	**0.004**	NC > a+mdMCI, AD

† *Mann-Whitney of verbal fluency measures between NC, MCI, and AD groups (Holm-Bonferroni corrected significant differences across groups was established in p = 0.008). Bold values of p means that the p-value was statistically significant*.

Pairwise comparison showed that the NC group significantly differed from all clinical groups in the number of new subcategories (Table 4). NC group performed significantly better than a+mdMCI and AD subjects also in the SVF variables of correct words, number of clusters and switching. There were no significant differences between aMCI, a+mdMCI and AD for all SVF variables, except a difference between aMCI and AD in the number of correct words produced (Table 4).

**Figure 1:**
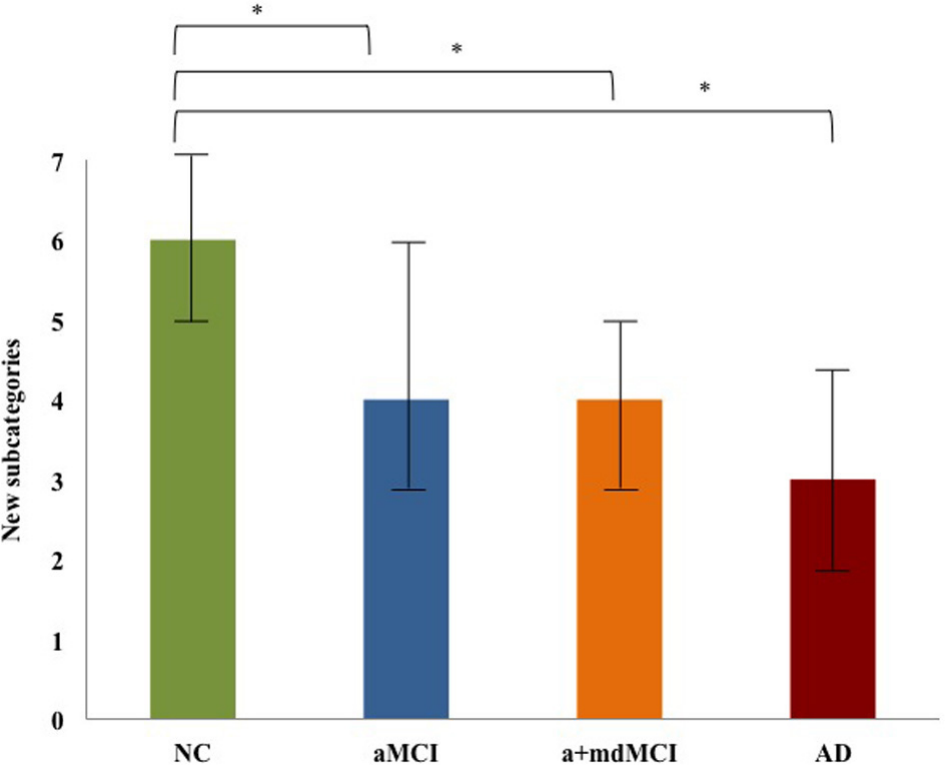
**New subcategories comparison among NC, aMCI, a-mdMCI, and AD groups**. ^*^Indicates the significant (*p* < 0.008;) difference between the groups.

The number of new subcategories is the only significant difference found between the NC and all the clinical groups, including the aMCI subtype (Figure 1). There were no differences between the four groups in the mean cluster size.

We found a marginal significant difference between the NC and AD groups in the number of developed clusters (*p* = 0.05;) and the mean size of the developed clusters (*p* = 0.05;).

## Discussion

The present study found that the number of animal subcategories produced in the SVF showed a significant decline in the amnestic MCI group compared to healthy controls. However, we did not observe significant differences in these measures between amnestic MCI, amnestic multiple-domain MCI and AD subjects. This may suggest that impairments in semantic memory are present in the early stages of the continuum of healthy cognitive aging, MCI, and dementia. In addition, we found that the number of clusters and switching were significantly reduced only in the amnestic multiple-domain and AD groups. These findings suggest that the latter subjects present with progressive difficulty to access the bulk of knowledge stored in semantic memory, possibly reflecting the presence of executive dysfunction in these subjects. Overall, these findings highlight the relevance of the assessment of other variables that can be extracted from an SVF task to understand the pattern of cognitive changes in the health cognitive aging, MCI and AD continuum.

The production of new subcategories was described as an alternative measure of semantic memory (March and Pattison, 2006). The generation of subcategories of animals depends on the knowledge of how a given animal relates to others or the shared attributes between them (e.g., pet animals or animals that are grown in farms) (Hoffman and Lambon Ralph, 2013). As animals in the same subcategory are closely related in the semantic memory system, once a person retrieves one animal it is easier to retrieve animals from that same subcategory following a structured semantic network (McClelland and Rogers, 2003). In our study we found that despite there were no significant differences in total of words produced in the SVF between controls and amnestic MCI participants, the latter showed a significant lower generation of animal subcategories. This finding suggests that the semantic memory system may be already disrupted in the earliest stages of the transition between normal cognitive aging and dementing disorders. Our findings are in line with the literature and highlight the importance of specifically evaluating semantic memory changes in addition to episodic memory in these subjects (Chan et al., 2001; Adlam et al., 2006; Joubert et al., 2008; Cuetos et al., 2009; Price et al., 2012).

It is worth noting that the switching measure were not significantly different between healthy controls and amnestic MCI participants, though were significantly lower in the amnestic multiple-domain MCI and AD. Switching is a measure of mental flexibility and indicates the ability to search and access novel information in the semantic memory system (Troyer et al., 1998; Nutter-Upham et al., 2008). These findings are in line with the theoretical decline in cognitive performance observed in the transition between amnestic MCI, multiple-domain MCI, and AD (Forlenza et al., 2009). Furthermore, our findings may suggest that the progressive decline in executive functions in subjects with MCI may indicate a higher risk of progression to AD (Rozzini et al., 2007).

The present results should be viewed in light of some limitations. We included a relatively small sample size what may have influenced the current analysis. Our sample has a low educational status. It is widely accepted that education is one of the main factors that influence the performance on a broad range of cognitive tests, including SVF (Radanovic et al., 2009). Also, education influences on how we store and retrieve information in the semantic memory (Reis and Castro-Caldas, 1997; Mathuranath et al., 2003). Therefore, we cannot exclude the possibility that some of the results are biased due to the educational status of our sample. Furthermore, our control group has a significant difference of gender distribution, which may be a limitation for the study. Nonetheless, previous studies did not find a significant effect of gender in clustering and switching (Troyer and Moscovitch, 2006; Weiss et al., 2006). Therefore, additional studies, including greater sample sizes, including subjects with more years of education and with a prospective design, are necessary to evaluate whether SVF variables (i.e., generation of new subcategories, clustering, and switching) can help to differentiate between healthy controls, MCI and AD subjects, as well as to identify those subjects of progressing to dementia upon follow-up. Our results also present overlapping values of SVF measures across the groups. Future research should address whether these SVF attributes can complement the neuropsychological assessment to differentiate older adults with distinct levels of cognitive impairment.

Our study highlights the importance of evaluating other variables from the SVF tests, like the generation of new subcategories, cluster and switching, in subjects with MCI and AD. The impairment of production of new subcategories indicates the presence of semantic memory impairment in amnestic MCI subjects. Difficulties in SVF switching measures harbinger the presence of executive dysfunction and the diagnosis of multiple-domain MCI and AD. Finally, these measures may help to identify those subjects at a higher for dementia.

### Conflict of interest statement

The authors declare that the research was conducted in the absence of any commercial or financial relationships that could be construed as a potential conflict of interest.
